# The impact of emotional labor on sleep-related worry among pediatric nurses in Chinese public hospitals: the mediating role of self-regulatory fatigue

**DOI:** 10.3389/fpubh.2026.1837829

**Published:** 2026-05-21

**Authors:** Yu Liu, Xiufang Zhao

**Affiliations:** 1 Department of Pediatric Intensive Care Unit Nursing, West China Second University Hospital, Sichuan University/West China School of Nursing, Sichuan University, Chengdu, Sichuan, China; 2 Key Laboratory of Birth Defects and Related Diseases of Women and Children (Sichuan University), Ministry of Education, Chengdu, Sichuan, China

**Keywords:** emotional labor, mediation analysis, pediatric nurses, self-regulatory fatigue, sleep-related worry

## Abstract

**Background:**

Pediatric nurses frequently engage in emotional labor in their interactions with children who have difficulty expressing themselves and with anxious families. This practice is associated with emotional numbness and burnout, and may negatively affect the quality of care. While pediatric nurses are a high-risk group for emotional labor, the intrinsic associations among emotional labor, self-regulatory fatigue, and sleep-related worry in this population remain unclear.

**Aims:**

This study aims to examine the mediating role of self-regulatory fatigue in the relationship between emotional labor and sleep-related worry.

**Method:**

This multicenter cross-sectional study recruited 604 pediatric nurses from various medical institutions across China. Data were collected using self-reported questionnaires, including a demographic questionnaire, the Emotional Labor Scale (ELS), the Self-regulatory Fatigue Scale (SRF-S), and the Anxiety and Preoccupation about Sleep Questionnaire (APSQ), which was used to assess sleep-related worry. Descriptive statistics, correlation analysis, univariate analysis, and multivariate analysis were performed using SPSS 27.0. Mediation analysis was conducted using the PROCESS macro (Model 4) for SPSS 27.0.

**Results:**

Emotional labor showed significant positive correlations with self-regulatory fatigue (*r* = 0.469, *p* < 0.01) and sleep-related worry (*r* = 0.460, *p* < 0.01). Self-regulatory fatigue was positively correlated with sleep-related worry (*r* = 0.394, *p* < 0.01). Mediation analysis indicated that self-regulatory fatigue statistically partially mediated the relationship between emotional labor and sleep-related worry, accounting for 26.9% of the total effect (based on 5,000 bootstrap samples; 95% CI for the indirect effect: [0.0493, 0.1243]).

**Conclusion:**

The findings indicate that, among pediatric nurses, emotional labor and sleep-related worry are positively associated, and this relationship is statistically mediated by self-regulatory fatigue.

## 1 Introduction

Pediatric nursing constitutes a uniquely critical component within the healthcare system. Unlike those in adult units, pediatric nurses must simultaneously manage children’s distress-induced crying and resistance, alongside parents’ anxiety and multiple treatment demands. Furthermore, pediatric nurses often face heavy patient loads, staffing shortages, and the need to balance the diverse demands of children, families, and team members, all of which contribute to substantial occupational stress and impaired sleep quality (1–3). This places pediatric nurses in a persistently high-stress work environment (4, 5). To address these challenges, nurses must continuously regulate their emotions—for example, by conveying friendliness and empathy through smiles, eye contact, and verbal communication to alleviate patients’ negative emotions and build trust (6, 7). This intensive, sustained process of emotional regulation, termed emotional labor, has become an indispensable core component of pediatric nurses’ daily work (4, 8). Research suggests that appropriate emotional labor can enhance nurses’ resilience. However, prolonged exposure to high levels of emotional labor significantly depletes psychological resources, heightens feelings of exhaustion, and is associated with negative outcomes such as professional burnout and depression (8–10).

When psychological resources depleted by emotional labor are not promptly replenished, nurses’ sleep health may be further compromised. Sleep-related worry is defined as an individual’s excessive worry about the consequences of poor sleep and fear of diminished control oversleep (11). Excessive worry about sleep may activate the autonomic nervous system, increase pre-sleep cognitive arousal, and create a vicious cycle of “worry-insomnia-more worry” (12, 13). Epidemiological studies have shown that the prevalence of sleep disorders among nurses is substantially higher than that in the general population. For instance, the prevalence reaches 78% among nurses in the UK (14) and 63.9% in China (15). Specifically, among pediatric nurses, a study of 274 nurses in eight tertiary hospitals found that 81.75% had poor sleep quality (PSQI ≥ 5), highlighting the urgent need to understand sleep-related worry in this population (1). Sleep disorders among nurses are associated not only with adverse health outcomes such as metabolic syndrome, cognitive impairment, cardiovascular disease, and cancer (15), but also with reduced quality of patient care and increased patient safety risks (16). A study of 1,831 Chinese nurses found that sleep-related worry not only directly diminishes job satisfaction but also indirectly weakens retention intentions by undermining work-related well-being, potentially contributing to adverse events (17). Sleep-related worry not only affects nurses’ personal well-being but has also been linked to increased medical errors, reduced attentional capacity, and higher rates of absenteeism, ultimately compromising patient safety and care quality. Therefore, elucidating the mechanisms underlying sleep-related worry among nurses and developing effective intervention strategies are essential for protecting their physical and mental health and ensuring high-quality nursing care.

Among the numerous factors associated with sleep-related worry, self-regulatory fatigue is considered a significant contributor (9, 18). Self-regulatory fatigue, a core concept within self-regulation theory, refers to the depletion of psychological resources following prolonged exertion of self-control efforts—such as emotional management, cognitive focus, and behavioral restraint—which results in diminished willpower and impaired regulatory capacity (18). According to the conservation of resources (COR) theory (19), individuals have a fundamental motivation to acquire, retain, and protect their psychological resources. When resources are continuously depleted without adequate replenishment, individuals may enter a “resource loss spiral,” triggering a series of negative psychological and behavioral responses. Emotional labor, as a typical resource-consuming process, requires nurses to continuously expend psychological resources. When this depletion reaches a critical threshold without timely recovery, self-regulatory fatigue may ensue. This state of fatigue may then impair nurses’ ability to regulate pre-sleep cognitive and emotional processes, making them more vulnerable to sleep-related worry. Thus, self-regulatory fatigue is theoretically positioned as a mechanism linking emotional labor to sleep-related worry (18, 20). Nurses experiencing self-regulatory fatigue exhibit significantly impaired emotional management and pre-sleep psychological regulation abilities, making it difficult to effectively cope with negative thoughts and anxiety before bedtime, which is likely associated with sleep-related worry (18, 20). Therefore, we hypothesize that self-regulatory fatigue mediates the relationship between emotional labor and sleep-related worry.

Existing studies have mostly examined the pairwise relationships among emotional labor, self-regulatory fatigue, and sleep-related worry separately. However, no study has integrated all three variables into a single mediation model specifically among pediatric nurses. Consequently, the mediating role of self-regulatory fatigue in the relationship between emotional labor and sleep-related worry in this population remains unclear. Pediatric nurses face unique emotional demands and high-intensity emotional labor, which may be associated with distinct mechanisms underlying sleep-related worry compared to nurses in other departments. Therefore, grounded in the conservation of resources (COR) theory, this study proposes a mediation model—“emotional labor → self-regulatory fatigue → sleep-related worry”—to examine the relationship between emotional labor and sleep-related worry among pediatric nurses and to test the mediating role of self-regulatory fatigue. Based on this analysis, the following hypotheses are proposed:

*H1*: Emotional labor is positively associated with sleep-related worry among pediatric nurses.

*H2*: Emotional labor is positively associated with self-regulatory fatigue among pediatric nurses.

*H3*: Self-regulatory fatigue mediates the relationship between emotional labor and sleep-related worry.

## 2 Methods

### 2.1 Study design

This study employed a cross-sectional design to investigate the relationships among emotional labor, self-regulatory fatigue, and sleep-related worry among registered pediatric nurses in public hospitals. Using a convenience sampling method, pediatric nurses from 10 public hospitals in Sichuan Province, Guangdong Province, Jiangsu Province, and Shanghai Municipality were recruited as study participants in February 2026. Sample size was calculated using the formula for quantitative research (21): $n={\left(u_{a/2}\sigma /\delta \right)}^{2}$ , the standard deviation (*σ*) for sleep-related worry scores among nurses was 4.49 (12), with a 95% confidence level (Z_(*α*/2) = 1.96) and a desired margin of error (*δ*) of 0.5, the required sample size was calculated as *n* = 310. Accounting for an anticipated 10% non-response rate, the final sample size was set at 345 participants.

### 2.2 Participant characteristics

Participants were registered nurses working in pediatric departments of public hospitals. The inclusion criteria were: (1) registered nurses currently working in pediatric departments of public hospitals; (2) at least 1 year of clinical experience in pediatrics; (3) voluntary participation in the study. The exclusion criteria were: (1) completion of the questionnaire in less than 3 min (indicating potential careless responding); (2) prior participation in similar questionnaire studies; (3) absence from clinical work for more than 6 months in the past year. A total of 640 questionnaires were distributed and 640 were returned (100% response rate). After excluding 36 questionnaires due to completion time of less than 3 min, 604 valid questionnaires were retained for the final analysis.

### 2.3 Measures

#### 2.3.1 Demographic questionnaire

This study used a self-developed questionnaire to collect sociodemographic information from pediatric nurses, including gender, age, marital status, parental status, educational attainment, professional title, years of service, department, hospital grade, frequency of night shifts, monthly income, self-rated health status, and job satisfaction.

#### 2.3.2 Sleep-related worry

This study employed the Anxiety and Preoccupation about Sleep Questionnaire (APSQ) to assess nurses’ sleep-related worry. Originally developed by Tang and Harvey (11), the scale was later adapted by Fröjmark et al. (22) and subsequently translated into Chinese by Shi et al. (23). The Chinese version has been validated and applied among nursing populations, demonstrating good reliability and validity (15, 24, 25). The scale consists of 10 items across two dimensions: concern about the consequences of poor sleep and concern about reduced control over sleep. Items are rated on a 5-point Likert scale ranging from 1 (“strongly disagree”) to 5 (“strongly agree”), with total scores ranging from 10 to 50. Higher scores indicate more severe sleep-related worry. The Chinese version of the scale has demonstrated good reliability, with a reported Cronbach’s *α* of 0.880. In the present study, the Cronbach’s α was 0.944, indicating excellent internal consistency within this sample.

#### 2.3.3 Emotional labor scale (ELS)

The Emotional Labor Scale was originally developed by Grandey (26) and later adapted into Chinese by Luo Hong (27). The Chinese version has been validated and applied among nursing populations, demonstrating good reliability and validity (28, 29). The scale consists of 14 items across three dimensions: surface acting (7 items), deep acting (3 items), and emotional expression demands (4 items). It employs a 6-point Likert scale ranging from 1 to 6, yielding total scores between 14 and 84, where higher scores indicate greater emotional labor among nurses. The Chinese version demonstrated a Cronbach’s *α* coefficient of 0.811, while the present study yielded a Cronbach’s α coefficient of 0.905, indicating high reliability of this instrument within the current sample.

#### 2.3.4 Self-regulatory fatigue scale (SRF-S)

The Self-regulatory Fatigue Scale (SRF-S) was originally developed by Nes et al. (30) and later adapted into Chinese by Wang et al. (31). The Chinese version has been validated and applied among nursing students, demonstrating good reliability and validity (32, 33). The scale consists of 16 items across three dimensions: cognitive control (6 items), emotional control (5 items), and behavioral control (5 items). Each item is rated on a 5-point Likert scale ranging from 1 (“strongly disagree”) to 5 (“strongly agree”). Total scores range from 16 to 80, with higher scores indicating greater self-regulatory fatigue. In the present study, the Cronbach’s *α* was 0.774, indicating acceptable internal consistency within this sample.

### 2.4 Data collection and quality control

Before distributing the questionnaires, we sent emails to the nursing departments and head pediatric nurses of the participating hospitals to explain the content and purpose of the survey. Questionnaires were distributed only after obtaining their consent. Data were collected electronically via Wenjuanxing,^1^ a widely used online survey platform in China that ensures data security and respondent anonymity. Participants accessed the survey by scanning a QR code. The first page of the questionnaire provided information about the study purpose, anonymity, confidentiality, and voluntary participation. Completion and submission of the questionnaire were considered as informed consent. This study was approved by the Ethics Committee of West China Second University Hospital, Sichuan University, which waived the requirement for written informed consent. The questionnaire included standardized instructions, required items, and was paginated to prevent missing responses. To ensure data integrity, each IP address was restricted to a single submission, and all responses were automatically anonymized upon export.

The research team monitored response quality in real-time to identify duplicates or anomalies. Following data export, two researchers independently cross-checked the dataset, excluding responses with implausible completion times, irrational answers, or logical inconsistencies. The resultant high-quality dataset was thus prepared for analysis.

### 2.5 Statistical analysis

Data management was conducted using Microsoft Excel, and statistical analyses were performed with SPSS version 27.0. Descriptive statistics were presented as frequencies (percentages) for categorical variables and as means ± standard deviations (SD) for continuous variables.

Normality of the continuous outcome variable (sleep-related worry) was assessed using the Shapiro–Wilk test, skewness, kurtosis (acceptable range: −2 to +2), and visual inspection of Q-Q plots and stem-and-leaf plots. The results indicated that the data were approximately normally distributed (skewness = −0.607, kurtosis = −0.193). Given the large sample size (*N* = 604), parametric tests were deemed appropriate.

Homogeneity of variance was tested using Levene’s test. For most variables, the assumption was met (all *p* > 0.05). For the two variables where the assumption was violated (work department and self-rated health), Welch’s correction was applied to adjust for unequal variances.

Group comparisons of continuous variables were conducted using independent-sample *t*-tests (for dichotomous predictors) or one-way analysis of variance (ANOVA) (for categorical predictors), provided that the assumptions of normality and homogeneity of variance were met, with Welch’s ANOVA applied when the homogeneity of variance assumption was violated. Associations between continuous variables were examined using Pearson’s correlation coefficient.

To assess potential common method bias, Harman’s single-factor test was conducted. The results showed that the first unrotated factor accounted for 31.16% of the total variance, below the recommended threshold of 40%, indicating that common method bias was unlikely to be a serious concern.

The hypothesized mediation model, in which self-regulatory fatigue mediates the relationship between emotional labor and sleep-related worry, was tested using the PROCESS macro for SPSS (Model 4) with 5,000 bootstrap samples. An indirect effect was considered statistically significant if its 95% bias-corrected bootstrap confidence interval did not include zero. The significance level for all tests was set at *α* = 0.05 (two-tailed).

## 3 Results

### 3.1 Descriptive analyses of main study variables (*N* = 604)

The mean scores for emotional labor, self-regulatory fatigue, and sleep-related worry among the 604 pediatric nurses were 58.62 ± 12.27, 46.69 ± 8.54, 34.57 ± 10.29, respectively (Table 1).

**Table 1:** Descriptive analyses of main study variables (*N* = 604).

Variables	Items	Min	Max	M ± SD
Total score of APSQ	10	10	50	34.57 ± 10.29
Worrying about the consequences of sleep	6	6	30	21.07 ± 6.30
Being unable to control sleep	4	4	20	13.50 ± 4.37
Emotional Labor Scale	14	14	84	58.62 ± 12.27
Self-Regulatory Fatigue Scale	16	16	80	46.69 ± 8.54

### 3.2 Demographic characteristics and univariate analysis of APSQ scores (*N* = 604)

A total of 604 pediatric nurses from 10 hospitals participated in this study. The majority of participants were female (95.4%, *n* = 576), and most were under 40 years of age (89.4%, *n* = 540), with 45.5% aged ≤30 years and 43.9% aged 31–40 years. Regarding educational attainment, 80.4% (*n* = 486) of participants held a bachelor’s degree or higher, including 76.3% with a bachelor’s degree and 4.1% with a master’s degree or above. In terms of professional title, 56.1% (*n* = 339) of participants held junior titles and 37.9% (*n* = 229) held intermediate titles. Most participants were employed in Grade III Class A hospitals (84.3%, *n* = 509) and held contract positions (80.6%, *n* = 487). More than half of the participants (57.5%, *n* = 347) rated their health status as “fair.”

Significant differences in sleep-related worry were observed across professional title, hospital grade, night shift frequency, job satisfaction, and self-rated health status (all *p* < 0.05) (Table 2).

**Table 2:** Personal characteristics and univariate analysis of APSQ (*N* = 604).

Demographic characteristics	Frequency	Percentage (%)	M ± SD	t/F	*p*
Gender				−0.657	0.511
Male	28	4.6	33.32 ± 10.29		
Female	576	95.4	34.64 ± 10.29		
Age (years)				2.730	0.066
≤30	275	45.5	34.19 ± 10.29		
31 ~ 40	265	43.9	35.49 ± 10.21		
41 ~ 50	64	10.6	32.38 ± 10.36		
Marital status				2.465	0.086
Unmarried	197	32.6	35.04 ± 10.53		
Married	387	64.1	34.10 ± 10.29		
Divorced	20	3.3	39.00 ± 5.97		
Education level				2.064	0.128
Junior College or below	118	19.5	35.47 ± 9.61		
Bachelor’s Degree	461	76.3	34.54 ± 10.39		
Master’s Degree or above	25	4.1	30.88 ± 10.99		
Professional title				3.204	0.041
Junior	339	56.1	34.39 ± 10.05		
Intermediate	229	37.9	35.42 ± 10.33		
Senior	36	6	30.86 ± 11.59		
Years of work experience				0.069	0.976
≤5 years	158	26.2	34.47 ± 10.62		
6 ~ 10 years	172	28.5	34.60 ± 9.96		
11 ~ 15 years	152	25.2	34.85 ± 10.70		
≥16 years	122	20.2	34.30 ± 9.90		
Hospital grade				3.312	0.037
Grade III, Level A	509	84.3	34.99 ± 10.02		
Grade III, Level B	66	10.9	31.56 ± 11.56		
Grade II and below	29	4.8	34.03 ± 11.10		
Having children				−0.945	0.345
Yes	351	58.1	34.23 ± 10.26		
No	253	41.9	35.04 ± 10.33		
Work department				2.272	0.086
Pediatric surgery	128	21.2	34.61 ± 8.34		
Pediatric internal medicine	325	53.8	33.80 ± 10.96		
PICU	76	12.6	35.43 ± 11.54		
Neonatology	67	11.1	37.36 ± 8.59		
Pediatric emergency	8	1.3	33.75 ± 8.26		
Employment type				2.338	0.097
Contract-based	487	80.6	35.01 ± 10.14		
Permanent staff (Bianzhi)	101	16.7	32.73 ± 10.93		
Labor dispatch	16	2.6	32.69 ± 9.92		
Night shift frequency (per month)				5.802	<0.01
0 times	138	22.8	31.91 ± 10.16		
1–4 times	176	29.1	34.16 ± 10.33		
5–8 times	209	34.6	35.67 ± 10.24		
≥9 times	81	13.4	37.15 ± 9.60		
Monthly income (CNY)				0.245	0.783
≤5,000	124	20.5	34.79 ± 11.25		
5,000–10,000	364	60.3	34.16 ± 10.33		
≥10,000	116	19.2	33.97 ± 9.90		
Job satisfaction				7.286	<0.01
Dissatisfied	4	0.7	40.50 ± 8.85		
In general	176	29.1	36.88 ± 9.76		
Satisfied	424	70.2	33.56 ± 10.36		
Self-rated health				52.967	<0.01
Relatively poor	88	14.6	42.41 ± 6.92		
Fair	347	57.5	35.13 ± 9.55		
Good	139	23	30.82 ± 9.49		
Excellent	30	5	22.47 ± 11.46		

### 3.3 Hierarchical regression analysis of APSQ among pediatric nurses

Based on univariate analysis results (*p* < 0.05), five demographic variables (night shift frequency, professional title, hospital grade, job satisfaction, and self-rated health) were selected as covariates. The measured data values were replaced, with the coding schemes for other predictor variables outlined in Table 3. Linear regression analysis was conducted with sleep-related worry as the dependent variable and emotional labor and self-regulatory fatigue as independent variables. The variance inflation factors (VIF) for all variables were below 1.3, indicating no multicollinearity issues. Hierarchical regression analysis was performed in three steps. Step 1 included demographic characteristics as control variables; Step 2 added emotional labor as an independent variable; Step 3 entered self-regulatory fatigue to examine the additional variance explained. The results of the hierarchical regression analysis are presented in Table 4.

**Table 3:** Assignment methods of independent variables.

Variable	Assignment of value
Professional title	Junior = 1; Intermediate = 2; Senior = 3
Hospital grade	Grade III, Level A = 1; Grade III, Level B = 2; Grade II and below = 3
Night shift frequency	0 times = 1; 1–4 times = 2; 5–8 times = 3; ≥ 9 times = 4
Job satisfaction	Dissatisfied = 1; In general = 2; Satisfied = 3
Self-Rated health	Relatively Poor = 1; Fair = 2; Good = 3; Excellent = 4

**Table 4:** Hierarchical regression analysis of sleep-related worry (measured by APSQ) among pediatric nurses (*N* = 604).

Variable	Model 1					Model 2					Model 3				
	*B*	*SE*	*β*	*t*	*p*	*B*	*SE*	*β*	*t*	*p*	*B*	*SE*	*β*	*t*	*p*
Constant	42.219	3.050	—	15.483	< 0.001	24.889	3.494	—	7.123	< 0.001	16.242	3.711	—	4.377	< 0.001
Professional Title	0.446	0.657	0.026	0.679	0.497	0.435	0.603	0.026	0.722	0.471	0.538	0.587	0.032	0.916	0.360
Hospital Grade	−0.620	0.751	−0.031	−0.826	0.409	0.032	0.691	0.002	0.046	0.963	0.128	0.673	0.006	0.191	0.849
Night Shift Frequency	1.091	0.411	0.104	2.658	0.008	1.030	0.377	0.098	2.734	0.006	1.086	0.367	0.104	2.960	0.003
Job satisfaction	−1.163	0.820	−0.054	−1.418	0.157	−0.907	0.752	−0.042	−1.205	0.229	−0.617	0.734	−0.028	−0.841	0.401
Self-Rated Health	−5.514	0.532	−0.394	−10.35	< 0.001	−4.281	0.502	−0.306	−8.533	< 0.001	−4.220	0.489	−0.302	−8.638	<0.001
ELS						0.313	0.029	0.373	10.665	< 0.001	0.229	0.032	0.274	7.188	<0.001
SRF-S											0.262	0.045	0.217	5.829	<0.001
*R* ^2^	0.195					0.324					0.360				
Adjusted R^2^	0.188					0.317					0.353				
F	28.907*					47.588*					47.897*				

*p < 0.001.

As shown in Table 4, after controlling for demographic variables, emotional labor was positively associated with sleep-related worry (*β* = 0.373, *p* < 0.001), explaining an additional 12.9% of the variance (ΔR^2^ = 0.129). Among the control variables, night shift frequency (*β* = 0.104, *p* = 0.008) and self-rated health (*β* = −0.394, *p* < 0.001) were significantly associated with sleep-related worry. When self-regulatory fatigue was entered in Step 3, it also showed a significant positive association with sleep-related worry (*β* = 0.217, *p* < 0.001), contributing an additional 3.6% of the variance (ΔR^2^ = 0.036). Notably, the effect of emotional labor was attenuated from *β* = 0.373 in Step 2 to *β* = 0.274 in Step 3, suggesting that self-regulatory fatigue partially mediates the association between emotional labor and sleep-related worry. The final model explained 36.0% of the variance in sleep-related worry (*R*^2^ = 0.360, adjusted *R*^2^ = 0.353, *p* < 0.001).

### 3.4 Correlation among major variables

To examine the relationships among emotional labor, self-regulatory fatigue, and sleep-related worry among pediatric nurses, Pearson’s correlation analysis was conducted. The results are presented in Table 5.

**Table 5:** Correlation among major variables.

Variables	1	2	3
1. Anxiety and preoccupation about sleep	1		
2. Emotional Labor Scale	0.460**	1	
3. Self-regulatory Fatigue Scale	0.394**	0.469**	1

^∗∗^*p* < 0.01, ^∗^*p* < 0.05.

Emotional labor was positively correlated with self-regulatory fatigue (*r* = 0.469, *p* < 0.01), indicating that higher levels of emotional labor were associated with greater depletion of self-regulatory resources. Emotional labor was also positively correlated with sleep-related worry (*r* = 0.460, *p* < 0.01), suggesting that nurses who engaged in more emotional labor reported higher levels of sleep-related worry. Additionally, self-regulatory fatigue was positively correlated with sleep-related worry (*r* = 0.394, *p* < 0.01), indicating that greater depletion of self-regulatory resources was associated with increased sleep-related worry.

### 3.5 Mediation analysis of emotional labor on sleep-related worry: the mediating role of self-regulatory fatigue

**Figure 1:**
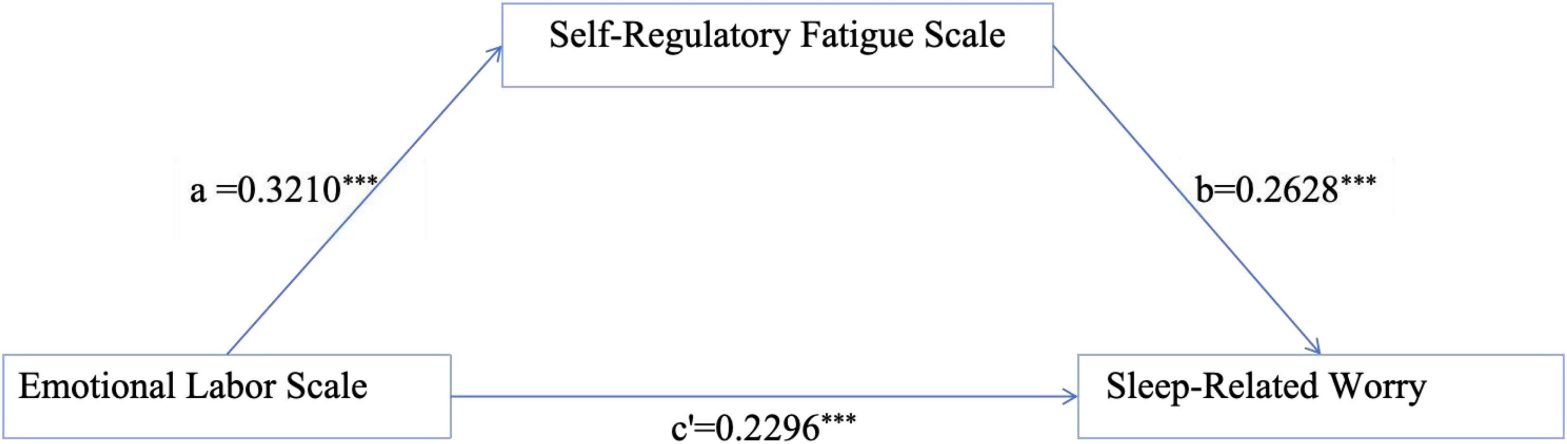
Mediating role of self-regulatory fatigue between emotional labor and sleep-related worry. ^***^p < 0.001. Covariates (night shift frequency and self-rated health) were controlled in the analysis.

To examine the mediating role of self-regulatory fatigue in the relationship between emotional labor and sleep-related worry among pediatric nurses, a mediation analysis was conducted (Figure 1). The results showed that emotional labor was significantly associated with sleep-related worry (*B* = 0.3140, *SE* = 0.0292, 95% CI [0.2567, 0.3712]). After including self-regulatory fatigue as a mediator, the direct association between emotional labor and sleep-related worry remained significant but was attenuated (*B* = 0.2296, *SE* = 0.0318, 95% CI [0.1672, 0.2921]), indicating partial mediation. The indirect association between emotional labor and sleep-related worry via self-regulatory fatigue was significant (*B* = 0.0843, *SE* = 0.0192, 95% CI [0.0493, 0.1243]), as the bootstrap 95% confidence interval did not include zero.

The results indicate that emotional labor is not only directly associated with sleep-related worry among pediatric nurses but also indirectly associated through self-regulatory fatigue. The indirect effect accounted for approximately 26.9% of the total effect, indicating that self-regulatory fatigue plays a significant mediating role in the relationship between emotional labor and sleep-related worry (Table 6).

**Table 6:** Mediation analysis of self-regulatory fatigue on the relationship between emotional labor and sleep-related worry.

Effect	*B*	SE	*β*	LLCI	ULCI	Proportion of total effect (%)
Total effect	0.3140	0.0292	0.3744	0.2567	0.3712	100
Direct effect	0.2296	0.0318	0.2738	0.1672	0.2921	73.1
Indirect effect	0.0843	0.0192	0.1006	0.0493	0.1243	26.9

## 4 Discussion

### 4.1 Descriptive statistics of emotional labor, self-regulatory fatigue, and sleep-related worry among pediatric nurses

This study found that pediatric nurses reported moderate to high levels of emotional labor, self-regulatory fatigue, and sleep-related worry. Specifically, the mean score for emotional labor was 58.62, indicating a moderately high level. This value is slightly higher than those reported in previous studies among nurses (34, 35). The mean score for self-regulatory fatigue was 46.69, also indicating a moderate to high level. This is slightly higher than the findings of Tai et al. (36) among 740 clinical nurses.

This discrepancy may be attributed to the unique characteristics of the study sample, which consisted exclusively of pediatric nurses. Pediatric nurses frequently encounter high expectations and demands from families regarding their children’s medical care, which contributes to substantial physical and psychological stress (5, 37). Prolonged exposure to such high-stress environments can deplete psychological resources, ultimately leading to self-regulatory fatigue (38).

The mean score for sleep-related worry among pediatric nurses was 34.57, indicating a moderately high level. This finding is consistent with Yang et al.’s (20) study of 330 anesthesiology nurses and is substantially higher than those reported in the general population (39, 40), suggesting that sleep-related worry is prevalent among nursing populations.

Notably, the score for the dimension “concern about the consequences of poor sleep” was significantly higher than that for “concern about reduced control over sleep.” This suggests that pediatric nurses are more concerned about the consequences and impacts of poor sleep on their daily work and life. This may be explained by two factors. First, the nature of nursing work demands high concentration, low error tolerance, and night shifts, all of which may contribute to sleep-related worry (18, 41).

Second, the nursing profession is predominantly female, and many nurses play central roles in their families. Night shifts encroach upon their rest time, making it difficult to balance family and work responsibilities, thereby increasing psychological stress and affecting sleep (41, 42).

This study further revealed that higher levels of sleep-related worry were observed among nurses with intermediate professional titles, those working in tertiary hospitals, those with night shift frequencies ≥9 times per month, those reporting low job satisfaction, and those with poor self-rated health. Previous studies have also identified associations between night shift frequency, self-rated health, and sleep-related worry among nurses (9, 18, 20, 41).

Therefore, nursing administrators may consider addressing sleep-related worry among nurses by reducing night shift frequency and ensuring adequate recovery time after night shifts. A cohort study (43) suggested that limiting total night shifts to fewer than 50 over a six-month period for nursing staff, and restricting long-term night shift workers to fewer than 39 shifts per six-month period, may help mitigate shift work-induced sleep disorders. However, these specific thresholds were derived from that study and were not directly tested in the present investigation; therefore, they should be interpreted with caution. Additionally, psychological support interventions could be considered for nurses with severe sleep-related worry to alleviate their distress (9).

### 4.2 Correlations among emotional labor, self-regulatory fatigue, and sleep-related worry in pediatric nurses

Pearson correlation analysis revealed significant positive correlations between emotional labor and sleep-related worry (*r* = 0.460, *p* < 0.01), between self-regulatory fatigue and sleep-related worry (*r* = 0.394, *p* < 0.01), and between emotional labor and self-regulatory fatigue (*r* = 0.469, *p* < 0.01). Previous studies have shown that emotional labor is positively associated with occupational burnout and turnover intention (44), and contributes to emotional exhaustion and resource depletion (45). Prolonged engagement in emotional labor depletes psychological resources, reducing nurses’ concentration and exacerbating self-regulatory fatigue (18).

Furthermore, heightened self-regulatory fatigue may foster negative thought patterns among nurses, thereby intensifying sleep-related worry (20). The significant correlations among these three variables provide an empirical basis for the subsequent mediation analysis.

### 4.3 Self-regulatory fatigue partially mediates the relationship between emotional labor and sleep-related worry among pediatric nurses

Mediation analysis showed that self-regulatory fatigue partially mediated the association between emotional labor and sleep-related worry among pediatric nurses (indirect effect = 0.0843, 95% CI [0.0493, 0.1243]), accounting for 26.9% of the total effect. This finding indicates that emotional labor is associated with sleep-related worry both directly and indirectly through the depletion of self-regulatory resources.

This study found that self-regulatory fatigue partially mediates the relationship between emotional labor and sleep-related worry among pediatric nurses. According to the conservation of resources (COR) theory, individuals’ psychological resources face both depletion risks and are driven by resource conservation motives; when individuals have sufficient resource reserves or access to external resource support, the negative effects of resource depletion can be buffered (19, 46). This suggests that the mechanism by which emotional labor is associated with sleep-related worry through self-regulatory fatigue does not operate in isolation but may be shaped by various individual and situational factors.

First, individual psychological characteristics—such as resilience, coping styles, and emotion regulation strategies—may moderate the effect of emotional labor on self-regulatory fatigue (18). Data from the present study showed that nurses who rated their health as “excellent” had significantly lower sleep-related worry scores (22.47) compared to those who rated their health as “fair” (35.13) or “relatively poor” (42.41). This suggests that positive psychological states may mitigate nurses’ negative emotions, reduce occupational burnout, and alleviate sleep-related worry (47).

Second, night shift frequency may influence this mediating pathway. In the present study, nurses with night shift frequencies of ≥9 times per month reported the highest levels of sleep-related worry (37.15). Night shift work constitutes both a physiological and psychological stressor. It not only disrupts normal circadian rhythms and sleep architecture but also limits opportunities for restorative activities such as rest and social interaction, thereby exacerbating nurses’ sleep-related worry (18, 45). This finding is consistent with the results of Zeng et al. (18). This suggests that nurses with frequent night shifts have greater difficulty recovering effectively after engaging in emotional labor, making them more susceptible to sleep-related worry via the pathway of self-regulatory fatigue.

Future research should further examine whether and how these factors moderate this mediating pathway, in order to gain a more comprehensive understanding of the mechanisms linking emotional labor to nurses’ sleep health.

## 5 Implications for practice

These findings provide important evidence for the development of tiered intervention strategies. First, for nurses with frequent night shifts, nursing administrators may consider environmental-level measures to reduce resource depletion. These include optimizing scheduling systems, extending recovery time after night shifts, and providing dedicated rest areas for night shift workers. Such strategies may help interrupt the pathway from emotional labor to sleep-related worry.

Second, for nurses with poor self-rated health, efforts could focus on enhancing personal resource reserves through regular health checkups, health promotion programs, and psychological support services, thereby improving their capacity to mobilize resources to cope with emotional labor. Additionally, self-rated health status could be considered as a sensitive indicator in routine mental health screening for nurses. Early attention and intervention could be directed toward nurses who rate their health as “fair” or “poor” to prevent them from entering a vicious cycle of self-regulatory fatigue due to resource depletion.

## 6 Limitation

This study has several limitations. First, the cross-sectional design precludes causal inferences; therefore, future longitudinal studies are warranted to establish temporal relationships. Specifically, although our mediation analysis is statistically consistent with the hypothesized model (emotional labor → self-regulatory fatigue → sleep-related worry), the cross-sectional nature of the data means that we cannot confirm the temporal ordering of these variables or rule out alternative mediation models.

Second, the majority of participants were female (95.4%), which limits the generalizability of the findings to male pediatric nurses.

Third, all variables were assessed using self-report questionnaires, which may introduce common method bias. However, Harman’s single-factor test indicated that the first unrotated factor accounted for 31.16% of the total variance, below the recommended threshold of 40%, suggesting that common method bias is unlikely to be a serious concern in this study.

Fourth, this study employed a convenience sampling method, recruiting participants from 10 public hospitals. Although this approach allowed for a relatively large sample size (*N* = 604), the sample may not be fully representative of all pediatric nurses in China, particularly those working in private hospitals, primary care settings, or different geographic regions. Therefore, our findings should be interpreted with caution, and future studies should consider probability sampling methods to enhance generalizability.

## Data Availability

The original contributions presented in the study are included in the article/supplementary material, further inquiries can be directed to the corresponding author.

## Footnotes

1
www.wjx.cn
